# A Population Model of Deep Brain Stimulation in Movement Disorders From Circuits to Cells

**DOI:** 10.3389/fnhum.2020.00055

**Published:** 2020-03-05

**Authors:** Nada Yousif, Peter G. Bain, Dipankar Nandi, Roman Borisyuk

**Affiliations:** ^1^School of Engineering and Computer Science, University of Hertfordshire, Hatfield, United Kingdom; ^2^Division of Brain Sciences, Imperial College Healthcare NHS Trust, Faculty of Medicine, Imperial College London, London, United Kingdom; ^3^College of Engineering, Mathematics and Physical Sciences, University of Exeter, Exeter, United Kingdom; ^4^Institute of Mathematical Problems of Biology, The Branch of Keldysh Institute of Applied Mathematics of Russian Academy of Sciences, Pushchino, Russia

**Keywords:** Parkinson’s disease, essential tremor, oscillations, computational modeling, beta band, gamma band

## Abstract

For more than 30 years, deep brain stimulation (DBS) has been used to target the symptoms of a number of neurological disorders and in particular movement disorders such as Parkinson’s disease (PD) and essential tremor (ET). It is known that the loss of dopaminergic neurons in the substantia nigra leads to PD, while the exact impact of this on the brain dynamics is not fully understood, the presence of beta-band oscillatory activity is thought to be pathological. The cause of ET, however, remains uncertain, however pathological oscillations in the thalamocortical-cerebellar network have been linked to tremor. Both of these movement disorders are treated with DBS, which entails the surgical implantation of electrodes into a patient’s brain. While DBS leads to an improvement in symptoms for many patients, the mechanisms underlying this improvement is not clearly understood, and computational modeling has been used extensively to improve this. Many of the models used to study DBS and its effect on the human brain have mainly utilized single neuron and single axon biophysical models. We have previously shown in separate models however, that the use of population models can shed much light on the mechanisms of the underlying pathological neural activity in PD and ET in turn, and on the mechanisms underlying DBS. Together, this work suggested that the dynamics of the cerebellar-basal ganglia thalamocortical network support oscillations at frequency range relevant to movement disorders. Here, we propose a new combined model of this network and present new results that demonstrate that both Parkinsonian oscillations in the beta band and oscillations in the tremor frequency range arise from the dynamics of such a network. We find regions in the parameter space demonstrating the different dynamics and go on to examine the transition from one oscillatory regime to another as well as the impact of DBS on these different types of pathological activity. This work will allow us to better understand the changes in brain activity induced by DBS, and allow us to optimize this clinical therapy, particularly in terms of target selection and parameter setting.

## Introduction

Deep brain stimulation (DBS) has been used over the last three decades to successfully treat the symptoms of a number of neurological and a few psychological disorders (Benabid et al., 1987; Mayberg et al., 2005). By far the most common disorders treated with DBS are movement disorders such as Parkinson’s disease (PD; Deuschl et al., 2006) and essential tremor (ET; Kupsch et al., 2006). PD is a movement disorder, with recognizable symptoms of tremor, slowness of movement and stiffness (Lees et al., 2009). ET is said to be the most common movement disorder, affecting up to one percent of adults over 40 years of age, and is characterized by an uncontrollable shaking of the affected body part (Brin and Koller, 1998; Deuschl et al., 1998; Louis et al., 1998). While the etiology of PD is known to be the loss of dopaminergic neurons in the substantia nigra, the impact of this on the basal ganglia networks are not fully understood, although a role for beta-band oscillations is widely accepted. On the other hand, the exact basis of ET remains unknown, and yet pathological neural oscillations in the thalamocortical-cerebellar network are also implicated in causing the symptoms.

The symptoms of both movement disorders can be treated with DBS, involving the implantation of electrodes into specific nuclei in the brain. For PD the target is typically the subthalamic nucleus (STN) and for ET the ventralis intermedius (Vim) nucleus of the thalamus. However, despite 70–80% of patients experiencing an improvement in their symptoms through DBS (Medtronics, 2013), the mechanisms through which this benefit is achieved remains elusive (Benabid, 2007; Kringelbach et al., 2007). Improved understanding of the physiological basis has been long sought, especially using computational modeling. Until recently however, the models used to study the impact of DBS have largely been focused on single neuron and single axon models with a high level of biophysical sophistication and detail (Davidson et al., 2016; Anderson et al., 2019; Howell et al., 2019). Our group has recently shown however, that the use of population or mean-field models can provide a great deal of insight into the mechanisms of both the underlying pathology as well as the mechanisms of DBS (Merrison-Hort et al., 2013; Yousif et al., 2017).

### Parkinson’s Disease and the Beta Band

In our mean-field study of PD, we examined oscillations in a multi-channel model. In that article, each channel consisted of an interconnected pair of STN and globus pallidus sub-populations and each of these channels was then connected, creating a multi-channel model (Merrison-Hort et al., 2013). We studied how the model behaved under both healthy and Parkinsonian conditions and showed that oscillations exist for a much wider range of parameters in the Parkinsonian case. We went on to discuss the link with experimental studies that have revealed details of network connectivity in Parkinsonian conditions. We also observed that the application of a DBS-like input caused the oscillations to become chaotic, and flattened the power spectrum.

### Essential Tremor and Oscillations

In another previous study, we used a combined experimental and again a mean-field theoretical approach (Yousif et al., 2017) to look at ET. Neural activity from the Vim was recorded intra-operatively *via* the DBS electrodes themselves, whilst simultaneously recording electromyographic activity from the contralateral affected limb(s). The thalamocortical-cerebellar network implicated in ET was modeled using the Wilson-Cowan approach. We found that the network exhibited oscillatory behavior within the tremor frequency range, as did our electrophysiological data. Applying a DBS-like input to the modeled network had the effect of suppressing the tremor band oscillations in the range of 4–5 Hz (Deuschl et al., 1998).

Together, these computational modeling studies show that the dynamics of the cerebellar-basal ganglia thalamocortical network support oscillations at frequency range relevant to movement disorders. Furthermore, the application of a DBS-like input into the modeled network disrupts such as pathological activity. We believe that this is an important way to study the impact of DBS on the human brain, and should be used in conjunction with experimental recordings of neural activity as well as with single-neuron biophysical modeling work.

### A Unified Network

In this article, we present new results from a combined model which exhibits Parkinsonian oscillations in the beta band, oscillations in the tremor frequency range, as well as oscillations in the gamma band which we term “healthy” (Beudel et al., 2015; Fischer et al., 2017). We find critical boundaries in the parameter space of the model separating regions with different dynamics. We go on to examine the transition from one oscillatory regime to another behavior and the impact of DBS on these two types of pathological activity. This approach will not only allow us to better understand the mechanisms of DBS but allow us to optimize the lengthy and difficult clinical process of parameter setting *via* trial and error, upon which the cited improvement in symptoms is reliant (Rizzone et al., 2001; Moro et al., 2002). For example, in future work, we could use this simplified approach to model the dominant frequency of oscillations in an individual’s local field potential data using population-level models, and use that personalized model to predict the effects of different DBS parameters on the dynamics of the network. This would allow the clinician to reduce the parameter space and search around the model predicted parameters. A recent study has reported using the Wilson-Cowan approach for understanding the onset of epileptic seizures (Wang et al., 2017). As multiple DBS parameter sets may well predict the same suppression of pathological oscillations in the model, this would also involve evaluating each predicted set for the clinical improvement in symptoms and any side effects, as well as considering the energy consumption of each setting. However, the intention would be that such an approach would reduce the time taken to select DBS parameters.

Furthermore, with the advent of electrodes with more contacts to improve selectivity (Buhlmann et al., 2011; Anderson et al., 2018), this process is becoming increasingly difficult. Multi-contact electrodes are a promising advance in the hardware for DBS, allowing directional stimulation and providing a wider field of stimulation due to the larger coverage. However, they also result in a higher dimensional parameter space. For example, one DBS four contact electrode has to have the following parameters defined: polarity of each contact (positive or negative), amplitude, frequency, pulse width. With eight contacts, at the very least we have to decide if twice the number of contacts are on/off and positive/negative. Hence the need for a theoretical understanding of DBS is particularly important at present.

## Materials and Methods

### Computational Model

**Figure 1:**
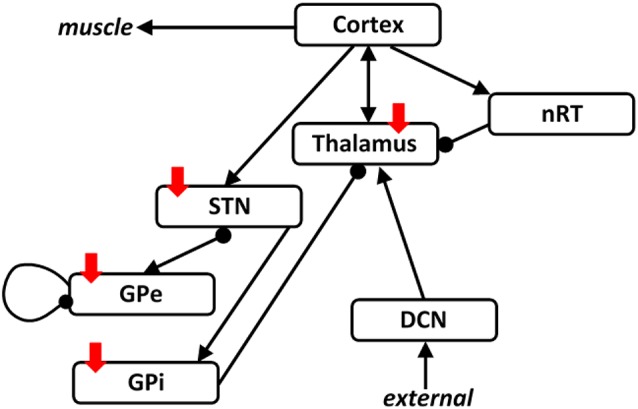
The thalamocortical basal ganglia network. The network we simulated here was based on our two previous studies looking at the thalamocortical network and the subthalamic nucleus (STN)-GPe network separately. Here, we combine the two networks with the addition of one population representing the GPi. The two networks are connected by including a cortical drive the STN, and an inhibitory output from the GPi to the thalamus. Arrows denote excitatory connections and round arrowheads denote inhibitory connections. Solid red arrows show the four brain regions targeted with deep brain stimulation (DBS).

As in our previous studies, we use a population representation of the combined thalamocortical basal ganglia network shown in Figure 1. This network is based on our previous studies, and the thalamocortical part is therefore based on the ET network (Yousif et al., 2017) and includes a cortical population, a cerebellar population and two thalamic populations. The basal ganglia part is derived from our previous Parkinson’s network study (Merrison-Hort et al., 2013) and includes a STN population and a population to represent the external part of the globus pallidus (GPe). In addition, here we include a population for the internal part of the globus pallidus (GPi), as this brain region is also implicated in movement disorders and particularly the pathology associated with PD. In order to connect the two networks, we include essential connections described in the literature, such as a cortical drive the STN, the so-called hyper-direct pathway. Also, the GPi is considered as the output of the basal ganglia and sends inhibitory output to the thalamus. As in our previous work (Yousif et al., 2017), the network receives an ascending drive *via* the cerebellar population to the thalamus.

As before, we used the Wilson-Cowan approach (Wilson and Cowan, 1972), which has been used widely to model populations of excitatory and inhibitory neurons connected into networks. The framework is based upon the assumption that neurons within a population are in close spatial proximity, and therefore ignore spatial interactions and only represent temporal dynamics. The activity of each population is represented by the proportion of cells that are firing action potentials per unit time.

As shown in Figure 1, we have an excitatory cortical population, two thalamic populations, the excitatory Vim nucleus and the inhibitory reticular nucleus (nRT), an excitatory population of cerebellar neurons, representing the deep cerebellar nuclei (DCN), the main output of the cerebellum, an excitatory population representing the STN, and two inhibitory populations representing the GPe and the GPi. Therefore, the model consists of seven first-order coupled differential equations, as shown here:

(1) $$\tau_{Cx}\frac{dE_{Cx}}{dt}=-E_{Cx}+(k_{e}-E_{Cx})\cdot Z_{e}(w_{1}E_{Th})$$

(2) $$\tau_{Th}\frac{dE_{Th}}{dt}=-E_{Th}+(k_{e}-E_{Th})\cdot Z_{e}(w_{2}E_{Cx}-w_{3}I_{nRT}+w_{4}E_{DCN}-w_{5}I_{GPi})$$

(3) $$\tau_{nRT}\frac{dI_{nRT}}{dt}=-I_{nRT}+(k_{i}-I_{nRT})\cdot Z_{i}(w_{6}E_{Cx})$$

(4) $$\tau_{DCN}\frac{dE_{DCN}}{dt}=-E_{DCN}+(k_{e}-E_{DCN})\cdot Z_{e}ext$$

(5) $$\tau_{GPe}\frac{dI_{GPe}}{dt}=-I_{GPe}+(k_{i}-I_{GPe})\cdot Z_{i}(w_{7}E_{STN}-w_{8}I_{GPe})$$

(6) $$\tau_{GPi}\frac{dI_{GPi}}{dt}=-I_{GPi}+(k_{i}-I_{GPi})\cdot Z_{i}(w_{9}E_{STN})$$

(7) $$\tau_{STN}\frac{dE_{STN}}{dt}=-E_{STN}+(k_{e}-E_{STN})\cdot Z_{e}(w_{10}E_{Cx}-w_{11}I_{GPe})$$.

Here, *E_i_* (*i* = Cx, Th, DCN or STN) and *I*_j_ (*j* = nRT, GPe or GPi) represent the number of active neurons in the relevant excitatory or inhibitory population at a given time. The strength of the connection between two populations is given by *w*_n_, where *n* = 1, 2…. 11. The value of this parameter represents the product of the average number of contacts per cell and the average postsynaptic current induced in the postsynaptic cell by a presynaptic action potential. Note that equation 4, for the DCN population, is independent of the dynamics of the other populations, and only provides an input into the Vim population. Therefore, in this model, the DCN population will tend to a stationary value and not oscillate.

Finally, the two functions *Z_e_(x)* and *Z_i_(x)* represent the proportion of cells firing in a population for a given level of average membrane potential activity *x(t)*. Previously, these functions were derived by assuming that the population has a distribution of neural thresholds and that all cells in the population have the same average level of membrane potential activity. Another approach can be to assume that the neurons within a population have the same threshold but varying numbers of afferent synapses. Either way, the result is that the response functions are monotonically increasing sigmoid functions, as shown here:

(8) Zp(x)=11+exp⁡(−bp(x−θp))−11+exp⁡(bpθp),

where, *p* represents *e* or *i*, *b_p_* and *θ_p_* are constants, and *x* is the level of input activity. We use the parameters given by Wilson and Cowan: *θ_e_* = 1.3, *b_e_* = 4, *θ_i_* = 2.0, and *b_i_* = 3.7. The maximum values of these response functions are given by the parameters *k_e_* and *k_i_*, where *k_e_* = 0.9945 and *k_i_* = 0.9994. The parameters *τ*_i_ represent the time constant of the change over time in the proportion of non-refractory cells that are firing in a population, in response to the change over time in the average membrane potential activity of the cells. This is typically set to be equal to the membrane time constant of the cells in the population, and normally in the range 10 ± 20 ms (Denham and Borisyuk, 2000). Here, all-time constants were set to 10 ms and unchanged for all simulations. The code generated for this study is available from the ModelDB website (accession number 261882).

### DBS Input

We simulated DBS of the network *via* the application of a high-frequency input to the GPe, STN or thalamus by modeling a simple square pulse as described here:

(9) $$DBS(t)=A\frac{4}{\pi }\sum_{n=1,3,5}^{1,001}\frac{1}{n}\sin(2n\pi ft),$$

where *A* is the amplitude of the input in arbitrary units (a.u.), *f* is the frequency and *t* is time. Hence the square wave is formed by summing the sin waves with the parameters above for odd values of *n*.

As previously described this change results in an additional term in the equation for the stimulated population, for example, the STN equation changed as follows:

(10) $$\tau_{STN}\frac{dE_{STN}}{dt}=-E_{STN}+(k_{e}-E_{STN})\cdot Z_{e}(w_{10}E_{STN}-w_{11}I_{GPe}+DBS)$$

### Numerical Details and Analysis

All simulations were run in Matlab *via* custom-written scripts. The ode solver “*ode23tb*” was used to numerically integrate the differential equations for each of the neuronal populations. A time step of 0.1 ms was used for simulations and the network activity was simulated for 1 s. The frequency of the resulting population activity was determined using the function “*pwelch*” on the second half of the simulated activity to avoid contamination by the transient activity at the start of the simulation. This function uses Welch’s method to calculate the power spectrum of a signal.

## Results

### Network Oscillations

**Figure 2:**
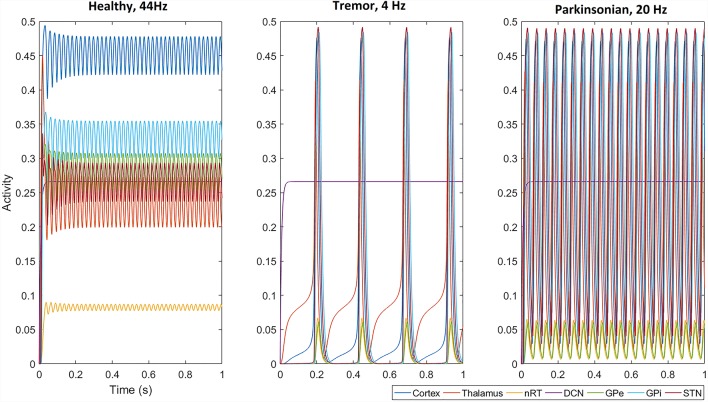
With a selected set of parameters (given in Table 1), all populations, except the deep cerebellar nuclei (DCN), oscillate in the gamma band at 44 Hz, the tremor band at 4 Hz and the beta band at 20 Hz. Note that the amplitude of oscillations across the network (apart from in the nRT and GPe) is smaller in the gamma band than in the two pathological activity states (tremor and beta).

**Figure 3:**
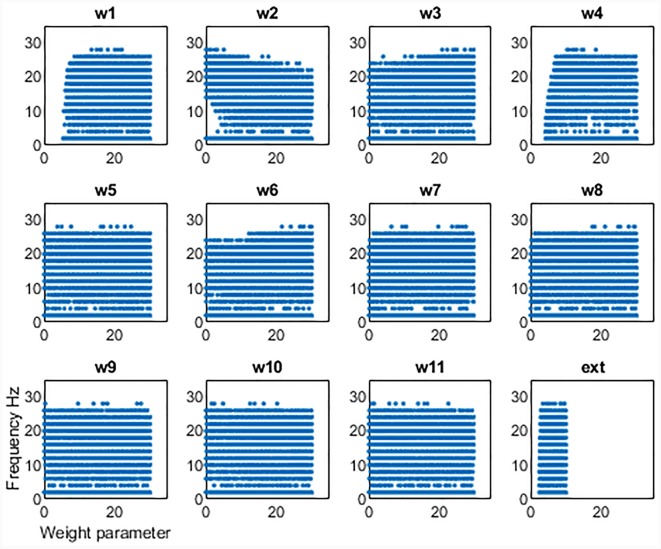
Results from 100,000 simulations of the network. Each point shows a simulation that leads to oscillatory network activity. The x-axis shows the value of the parameter for that simulation and the y-axis the frequency of the activity.

We first examined the network activity as a whole, to understand the dynamics of this thalamocortical-basal ganglia network. Hence we simulated the network and explored the connection weights parameter space to find the regions which produced oscillatory activity in the frequency ranges we were particularly interested in. Namely, gamma-band healthy oscillations (>30 Hz; Beudel et al., 2015; Fischer et al., 2017), the tremor band (Deuschl et al., 1998) as we previously observed in our EMG-LFP data (Yousif et al., 2017), and the beta band Parkinsonian oscillations (Hammond et al., 2007). Examples of these three classes of oscillations are shown in Figure 2 and discussed in more detail below. Overall, we found that the network readily oscillated for a large region of the parameter space. Figure 3 shows the results of 100,000 simulations, with randomly assigned parameter weights between 0 and 30, and a randomly assigned ascending drive (ext) between 0 and 10. We found that this number of simulations was sufficient to observe a variety of oscillatory behavior. For each parameter, a point represents a simulation resulting in the oscillatory network activity, and the y-axis shows the frequency of that activity. It is clear that the network is capable of oscillating throughout the parameter regions of interest.

This set of results demonstrates a key result. The network will not oscillate if some key connections are cut. First, the thalamic ascending drive to the cortex is necessary to achieve oscillations. Second, the ascending input into the DCN is also necessary for oscillations to exist. However, the remainder of the connections can be equal to zero and the network will still oscillate. Hence the only critical “connections are those two ascending connections. It also appears that there is some dependence of frequency on the corticothalamic feedback connection (w_2_), as at lower values of this connection weight, the frequency of oscillations can increase. The opposite may be said for the inhibitory reticular input to the thalamus (w_3_), as at greater strengths of this connection, higher values of frequency can be seen. However, to unpick these patterns in more detail, we focussed on the tremor bands activity in more detail, varying individual parameters one by one and then pairwise to better understand the relationship between them.

### Transition to Tremor Band Activity

First, we set up the network with a set of parameters that displayed gamma-band oscillations (Figure 2) which we set to be our healthy oscillations. We then made minimal changes to the parameters to move the oscillatory activity into the tremor band of 4–5 Hz and used this as our baseline for tremor activity. These changes involved increasing the corticothalamic feedback parameter (w_2_), decreasing the DCN drive to the thalamus (w_4_) and decreasing the STN to GPe connection weight (w_7_). The parameters are given in Table 1 and remained unchanged for subsequent simulations. With this set of baseline parameters, all of the populations in the neuronal network, except the DCN, oscillate at 4 Hz. Interestingly, the amplitude of the oscillation, which represents the proportion of neurons active in a population, is greater for most populations in the tremor state than in the healthy state. The amplitude of oscillations in the tremor state also varied across the population and was greatest in the STN population, followed by GPi, Cortex, Thalamus, nRT and GPe with the smallest. The oscillations were lead however, by the thalamus, followed by Cortex, nRT, STN, GPe and GPi at the end.

**Table 1:** The 11 connection weights between neuronal populations and the external ascending drive parameters are listed here.

Connection	Weight	Polarity	Healthy state parameters	Tremor band parameters	Beta band parameters
Th -> Cx	w1	+	20	20	20
Cx -> Th	w2	+	5	12	5
nRT -> Th	w3	−	8	8	8
DCN -> Th	w4	+	25	9	20
GPi -> Th	w5	−	15	15	15
Cx -> nRT	w6	+	5	5	5
STN -> GPe	w7	+	19	5	5
GPe -> GPe	w8	−	5	5	5
STN -> GPi	w9	+	15	15	15
Cx -> STN	w10	+	20	20	20
GPe -> STN	w11	−	20	20	20
ext -> DCN	ext	+	3.42	3.42	3.42

*For each connection, the label used throughout the text and figures is given, as well as the polarity of the connection, i.e., whether it is excitatory (+) or inhibitory (-), as well as three sets of parameters which result in healthy gamma band, tremor band and beta band activity*.

**Figure 4:**
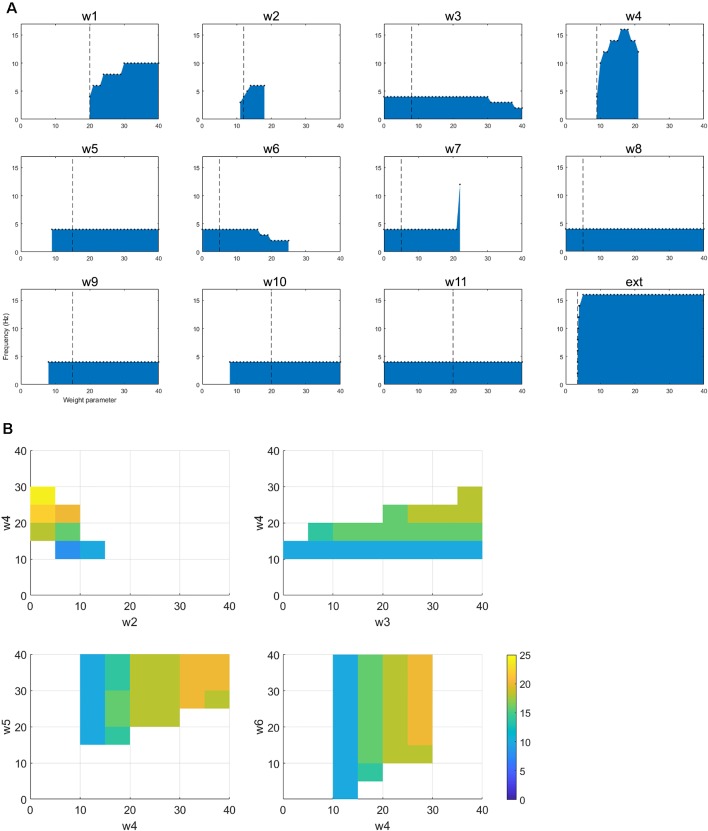
**(A)** The figure shows how changing a single parameter affects the presence of oscillatory activity and the frequency of that activity when the network is oscillating in the tremor band.** (B)** For the selected tremor band activity, we performed pairwise parameter manipulations keeping the rest of the parameters at the values given in Table 1. Here, the plots show the pair-wise combinations which resulted in oscillatory activity and the values of the parameters for those simulations.

In order to better understand the dynamics of the network when in the tremor band, we first varied each parameter individually within the range 0–40, in order to observe the impact on the oscillatory activity. All other parameters remained at the value in Table 1. Figure 4A shows the results from this set of simulations. First, we note that in this particular instance of the network most connections are required for this tremor band oscillatory activity to be maintained. The exception to this is w_3_, w_6_, w_8_ and w_11_. The first two of these are the connections made by and to the nRT and the latter two are the connections made by and to the GPe. This may indicate that these structures are superfluous to the oscillations, however, the frequency of the oscillations is certainly altered by the connections of the nRT. In this network state, however, the GPe does not appear to have much effect on the oscillations in the tremor band. However, if we look closely at the plot for w_7_, which is the weight of the connection from STN to GPe, we see that if this weight is less than or equal to 21, the frequency of oscillations is unchanged from the default of 4 Hz. At 22 however, there is a dramatic shift to 12 Hz activity. Hence the role of the GPe may be significant in shifting the activity of the network between oscillatory states.

Looking at changes in the frequency of the oscillations more closely, we see that three more parameters have some impact on this: w_1_, w_4_, and the ext parameter. These are all the ascending inputs in the network: external input to the DCN, DCN to thalamus and thalamus to the cortex. These parameters are able to increase the frequency of the oscillations out of the tremor band towards the beta band. Interestingly, w_4_ is the only one of this subset, which has an upper limit (within the range tested here), with w_1_ and ext parameter not displaying such a limit.

This change in activity with parameter variations was also examined in a pairwise fashion. Each parameter was varied in the range 0–40, with each other parameter, to test for the presence of oscillations and the frequency of that activity. Figure 4B shows four of the most interesting results from this set of simulations. All four of these plots show a similar pattern, which is that increasing the ascending drive to the thalamus (w_4_: DCN -> Th) can drive the oscillation into a higher frequency band if this is paired with a corresponding increase in inhibition (*via* GPi->Th w_5_, nRT -> Th w_3_ or Cx->nRT w_6_) to the thalamus or a decrease of cortical excitation to the thalamus (w_2_). Interestingly then, this network can readily switch into beta-band activity with a number of single parameter manipulations (Figure 4A) or multiple parameter variations (Figure 4B).

### Transition to the Beta Band

Hence, we selected one such change in parameters to result in a high beta band oscillation and used this as our basis for examining this frequency of oscillation in the network. We decreased w_2_ and increased w_4_, resulting in a set of parameters given in Table 1. The network oscillated readily at 20 Hz with this set of parameters. All other parameters remained unchanged from the tremor band state and all parameters were kept at these default values for the remainder of the simulations. Figure 2 shows this 20 Hz activity was consistent across the network, except the DCN, and showed a similar pattern to the tremor band activity. The STN shows the highest amplitude and the thalamus leads the oscillations.

**Figure 5:**
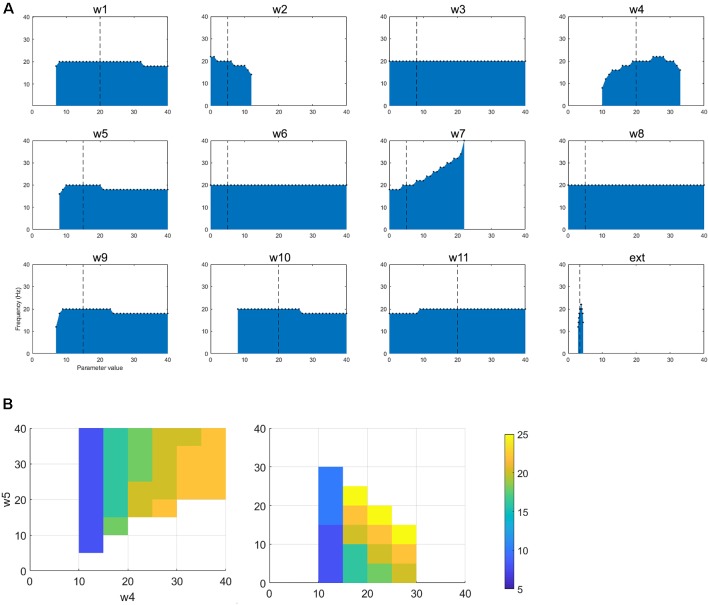
**(A)** The figure shows how changing a single parameter affects the presence of oscillatory activity and the frequency of that activity when the network is oscillating in the beta band. **(B)** For the selected betaband activity, we performed pairwise parameter manipulations keeping the rest of the parameters at the values given in Table 1. Here, the plots show two pair-wise combinations that resulted in oscillatory activity and the values of the parameters for those simulations.

Once again, the single parameter variations from the default set of parameters show an interesting set of results (Figure 5). First, w_1_, w_4_, w_5_, w_9_, and w_10_ cannot be set to zero if oscillations are to be maintained. w_2_ can only hold a low range of values (including zero). w_7_ (STN to GPe) can drive oscillations up to a higher frequency (40 Hz). Ext can only have a small range of values. As for the tremor band oscillation, we also examined the impact of parameter changes in a pairwise fashion. Figure 5B shows two of the most interesting results from this set of simulations. Both plots show that decreasing the ascending drive to the thalamus (w_4_: DCN -> Th) can move the oscillation into a lower frequency band if this is paired with a corresponding decrease in inhibition (*via* GPi->Th w_5_, or STN->GPe w_7_). Hence this is consistent with the results for the tremor band pairwise variations.

### Modeling DBS Input

**Figure 6:**
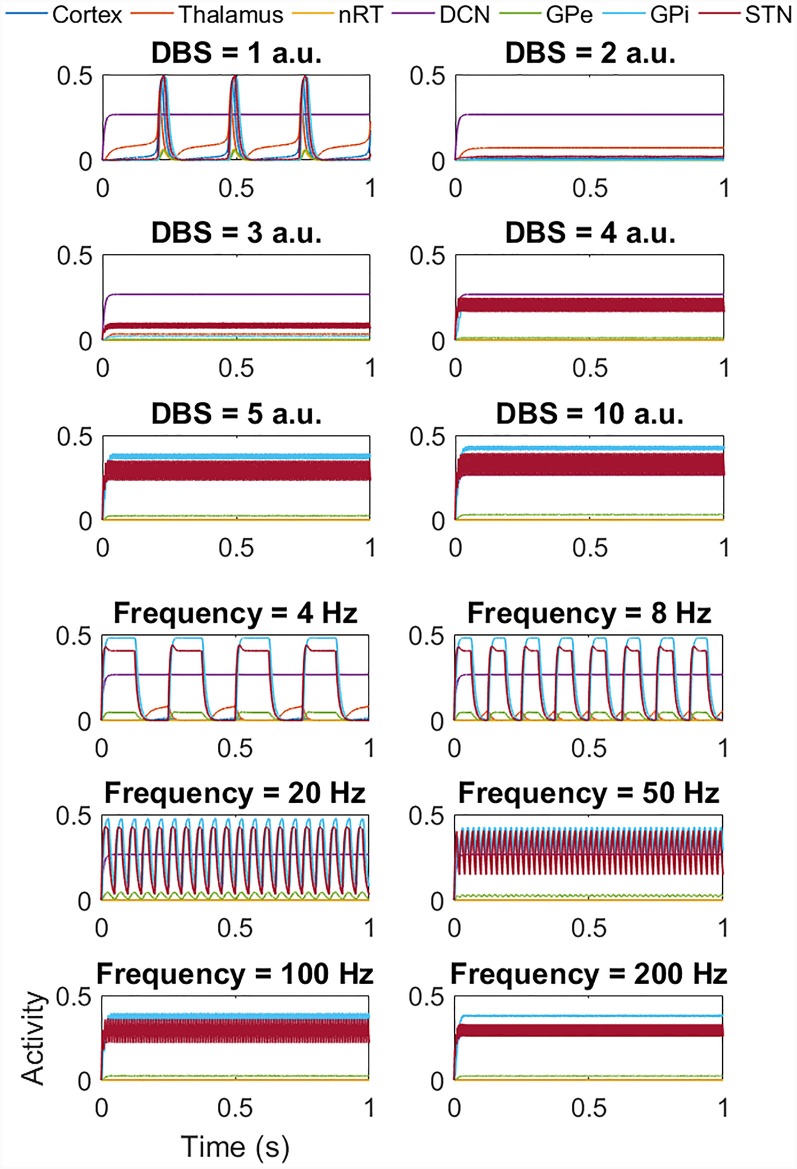
Impact of STN DBS on the tremor band activity. The top six plots show the effect of increasing the DBS amplitude (with DBS frequency = 120 Hz) from 1 a.u. to 5 a.u. and then 10 a.u. As the DBS amplitude increases, the frequency of oscillations first decreases (as evident in Amplitude = 1 a.u.) and then the high amplitude low-frequency activity is replaced by low amplitude high-frequency activity. The lower six plots show the effect of DBS frequency (with DBS amplitude = 5 a.u.). At low frequencies, there is a reduction in amplitude and an increase in the frequency of the oscillations. From 50 Hz onwards this effect is clear. It is important to note that at frequencies greater than 100 Hz, the network activity is oscillatory and all populations except the DCN oscillate at the stimulation frequency.

DBS was applied to the network, *via* a square pulse as described above. With DBS to the STN and the network displaying tremor band activity, Figure 6 shows that as the DBS amplitude increases, the frequency of oscillations first decreases (as evident in Amplitude = 1 a.u.) to approximately 3 Hz activity. As the DBS amplitude increases further, the high amplitude low-frequency network activity is replaced by low amplitude high-frequency activity. The lower six plots in Figure 6 go on to show the effect of changing the DBS frequency, with the DBS amplitude set at 5 a.u. At low DBS frequencies, there is a reduction in the amplitude of the oscillations, and an increase in the frequency of the oscillations. From 50 Hz onwards this switch from low frequency to high-frequency network activity is complete. We also applied DBS to the thalamic population, the GPi and the GPe populations to mimic clinical practice. In the cases of thalamic and GPi stimulation, there was a similar effect. However, for the thalamus, the underlying network oscillation was only suppressed at 5 a.u. and above. Conversely for the GPi, DBS stimulation from 1 a.u. suppressed the network oscillation. The GPe population also showed a suppression of the tremor band oscillation when DBS was applied to it, but in this case, the rest of the network continued to oscillate at 4 Hz.

**Figure 7:**
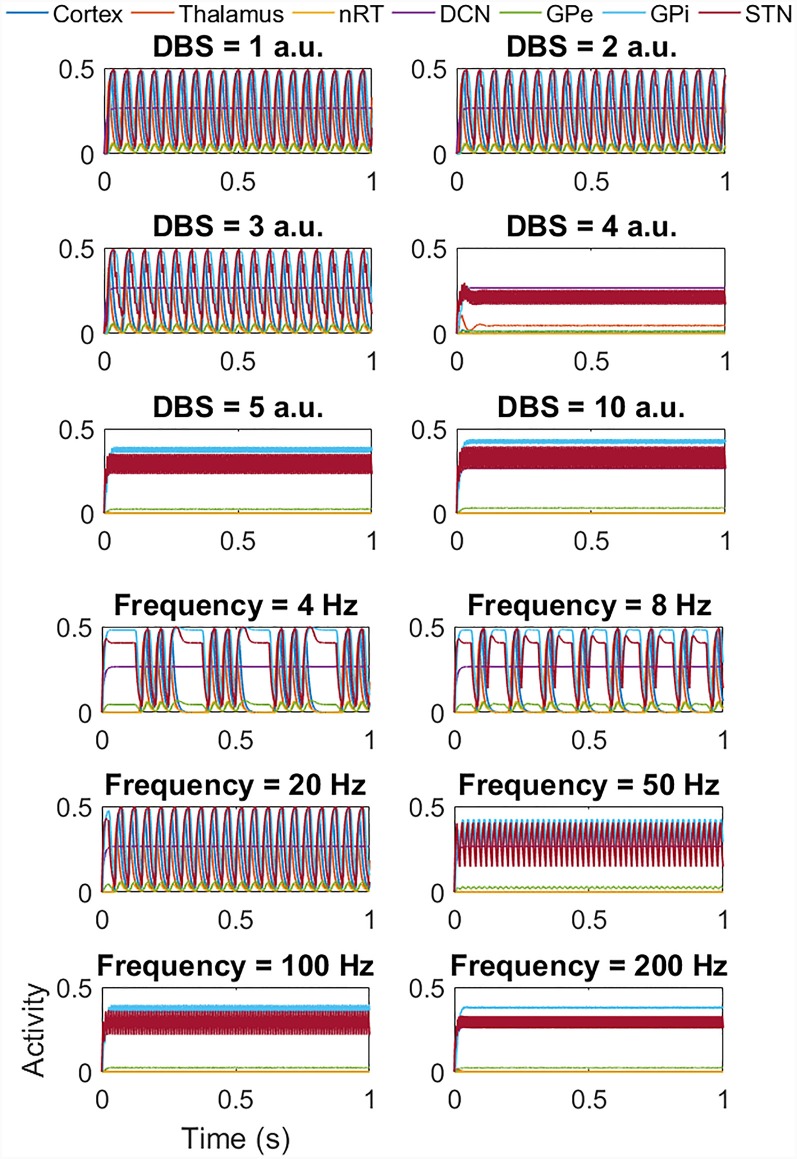
Impact of STN DBS on the beta band activity. The top six plots show the effect of increasing the DBS amplitude (with DBS frequency = 120 Hz) from 1 a.u. to 5 a.u. and then 10 a.u. As the DBS amplitude increases, there is little change in the frequency or amplitude of oscillations but at 4 a.u. and above, the high amplitude low-frequency activity is replaced by low amplitude high-frequency activity as for the tremor band activity. The lower six plots show the effect of DBS frequency (with DBS amplitude = 5 a.u.). At low frequencies, there is no change in amplitude, but a reduction in the frequency of the oscillation, with the activity appearing to occur in bursts. Above 50 Hz the high amplitude low-frequency activity is replaced by low amplitude high-frequency activity. a.u.: arbitrary units.

Interestingly, for the beta band activity, the application of DBS shows a similar but slightly different effect, as seen in Figure 7. At low DBS amplitude, there is little change in the amplitude or frequency of the network activity, but from 4 a.u. and above, we once again see a switch from high amplitude low-frequency network activity to low amplitude high-frequency activity as for the tremor band simulations. The lower six plots again show the effect of DBS frequency, this time with DBS amplitude = 5 a.u.). In this case, at low frequencies, there is no change in the amplitude of the network activity, but we do see a reduction in the frequency of the oscillation, with the activity appearing to occur in bursts. Above 50 Hz the high amplitude low-frequency activity is replaced by low amplitude high-frequency activity. We once again applied DBS to the thalamic population and the GPi and GPe populations and saw a similar pattern in thalamic DBS (although occurring at a higher amplitude of 5 a.u. and above) and GPi DBS. Once again for DBS of the GPe, there was a suppression of the beta band oscillation in the GPe population, but the network continued to oscillate at 20 Hz. Interestingly however, the GPe also showed the bursting behavior at low DBS frequencies seen in the STN, thalamus and GPi cases.

## Discussion and Conclusion

The pathological changes that occur in the human brain as a result of PD and other movement disorders may be well understood in terms of the changes in neurophysiology. For example, it is well known that in PD there is a loss of dopamine neurons in the substantia nigra (Dauer and Przedborski, 2003), however, in ET, the cause of the pathology is less understood. However, one overriding feature of disease is that brain signals, either from clinical recordings (Kühn et al., 2006; Brown, 2007; Beudel et al., 2019; Lofredi et al., 2019) or animal models of movement disorders (Tachibana et al., 2011; Deffains and Bergman, 2019) have been shown to demonstrate oscillatory activity. While the presence of such pathological oscillations is well documented, the generation and propagation of such neural activity remain poorly understood. To improve this understanding, particularly in PD and ET, and critically the impact of DBS on this pathological activity, we constructed a population-level neuronal model of the basal ganglia, thalamocortical network. This model builds on and extends our previous studies which focussed on PD (Merrison-Hort et al., 2013) or ET (Yousif et al., 2017) independently, and aimed to address how different types of oscillations are supported and affected by the dynamics of the underlying network. The main feature of this modeling approach, based on the Wilson-Cowan approach, is its simplicity and lack of detail. This simplicity is an intentional feature as the aim of the model is to look at only the dynamics of the network without reliance on other physiological properties, however, it is important to note that this also imposes some limitations, such as the fact that all populations in the network display the same frequency activity during oscillatory regimes.

Our model showed that this network readily demonstrated a wide range of oscillatory activity up to 44 Hz, and in particular in the frequency bands which have been identified as of importance to movement disorders. This wide range in itself interesting as the network can represent, for example, steady-state non-oscillatory activity, different frequencies of pathological and physiological oscillations. It is important to note however, that the transitions to other frequency bands were achieved from a single set of parameters which resulted in gamma activity. We have examined other gamma states which were identified in our simulations, and it was also possible to transition to tremor and beta states from those parameters. Specifically, we were interested in the low-frequency tremor band ~4–8 Hz for both ET and PD tremor (Raethjen et al., 2007; Reck et al., 2009; Tass et al., 2010; Pedrosa et al., 2012), the beta band which has been hypothesized to be hypokinetic in PD, ranging from 13 to 30 Hz (Jenkinson and Brown, 2011; Little and Brown, 2014) and the gamma band which has been hypothesized to be pro-kinetic in PD (Bočková and Rektor, 2019). Critically, when surveying the parameter space we found that only two connections always had non-zero weights for such oscillatory activity to be present. First, the thalamic input into the cortex and secondly the cerebellar input into the thalamus. Hence the driving or ascending connections of the network must be intact for the pathological oscillations to exist, which is consistent with our previous work (Yousif et al., 2017).

Focussing on each frequency band in turn, the reliance of the tremor band oscillations (4 Hz activity) on the connections of the network was showed a different pattern to when we look at the full range of oscillatory frequencies. Once again, the ascending connections were required for the oscillations to be maintained, however, in addition, the cortical and pallidal inputs to the thalamus and the STN to GPi connections were also required. Hence, it appears that the thalamus acts as a hub for the support of these low-frequency oscillations. This is consistent with the clinical practice of thalamotomy, thalamic DBS and MRI guided focussed ultrasound of the thalamus for relieving tremor (Bretsztajn and Gedroyc, 2018; Halpern et al., 2019). Furthermore, it is also supported by the targeting of zona incerta (ZI) during STN DBS (Yousif et al., 2012), a region highly connected with the thalamus.

We were interested to determine how the change to beta-band activity from the tremor band could be achieved in the network, particularly given a recent study which postulates that tremor band oscillations are a by-product of suppression of beta-band oscillations (Müller and Robinson, 2018). We found that one route to achieving this switch was by decreasing the cortical drive to the thalamus and increasing the cerebellar drive to the thalamus. Hence, there is overall no change to the excitatory inputs to the thalamus, but a change in the balance of ascending and descending inputs, which in turn affects the timing of the excitatory input to the thalamus. While the connections needed for beta oscillations remain similar to those in the tremor band, one connection could now be severed and oscillations would continue, which is the corticothalamic input. The driving thalamocortical connection is critical for oscillations to persevere, and even when looking in two-dimensional parameter space we did not find a combination of parameters that would compensate for severing the thalamocortical connection. However, the feedback connection from cortex to thalamus is not essential. Interestingly, the role of beta in PD and normal movement is a topic of great discussion in the literature (Little and Brown, 2014), and it remains debated whether beta is truly a hallmark of pathology.

Interestingly, recent work has shown that tremor band oscillations emerge in a model oscillating in the beta band when the GPe to STN and cortex to STN coupling strengths were reduced (Müller and Robinson, 2018). The STN and GPe network has been previously studied extensively, by ourselves (Merrison-Hort et al., 2013) and others (Gillies et al., 2002; Terman et al., 2002), with the hypothesis that it is critical for Parkinsonian oscillations. We found that in both the tremor state and the beta state, this connection was able to singlehandedly and dramatically change the frequency of the oscillatory activity. In addition, this was despite the fact that the oscillations could persist in the absence of this connection. Hence, we also found that the connection between STN to GPe provided one route to switching the frequency of oscillations in the network, not only from tremor to low beta but then from beta to gamma oscillations.

The application of DBS to the network was the main focus of the current study. We applied DBS in both states of the network, at different amplitudes, at different frequencies and to four populations in turn: the thalamus, commonly targeted for treating ET (Benabid et al., 1987), the STN, a typical target for PD (Wichmann and DeLong, 2011), the GPi and the GPe which are also used as a targets for PD (Burchiel et al., 1999; Vitek et al., 2012). When the network was exhibiting tremor band activity, we found that the effect of a DBS like input was effective at suppressing the low frequency, high amplitude pathological activity and replacing it with low amplitude high-frequency activity. This is consistent with our previous work (Yousif et al., 2017) and other reports (Hassler et al., 1960) as well as studies which have shown that STN DBS increases pallidal firing rates and regularises neuronal activity to a bimodal firing rate distribution (Hashimoto et al., 2003).

At frequencies lower than those typically used therapeutically, DBS appeared to first lower the frequency of the network activity and then with increasing DBS frequency, suppressed the amplitude and increased the pulse width of the neural activity, which may agree with the empirical observation that low-frequency DBS intensifies pathological tremor (Fogelson et al., 2005; Oza et al., 2018). Such a hypothesis requires simultaneous DBS and recording to be fully understood. Furthermore, previous work has proposed that DBS may act to replace the irregular abnormal activity with more regular firing using biophysical models (Rubin and Terman, 2004). Similarly, the pathological high amplitude oscillations in our model may act as a block to the normal activity of the network, resulting in symptoms of the disease. The application of DBS and the switch to the low amplitude high-frequency oscillations may allow the network to revert to normal functioning such as relaying information through the thalamus. Finally, we found that the application of DBS during tremor band oscillations had a similar effect regardless of the target population, except to the GPe, which hypothesizes that STN and GPi DBS could also change tremor band neural activity. A recent study has shown that STN and GPi DBS showed no statistical difference in suppressing tremor (Wong et al., 2019). The thalamus is typically used as a DBS target for ET rather than PD tremor, and GPe stimulation did not show full tremor suppression throughout the network in our model. Our predictions suggest further work is needed to compare the efficacy of each target on different symptoms.

Interestingly, recent work has shown that the phase of stimulation could be particularly significant for applying high-frequency bursts of DBS (Cagnan et al., 2017) phase-locked to a patient’s tremor. We ran a set of simulations to investigate the effect of phase when stimulating the network but found that a stimulus was phase-locked to the network oscillation had the same effect as a stimulus that was not. A similar effect was seen for the beta band network oscillations. This may indicate that the phase-dependent results are dependent on the physiology of the neuronal networks not explicitly represented by the network here.

Electrophysiological recordings have shown that DBS acts to attenuate beta-band oscillations (Quinn et al., 2015). Interestingly, we found that DBS during beta-band oscillations showed a different effect on the network activity to the tremor band case. First, the amplitude required to suppress beta-band oscillations was higher than in the tremor band case. Second, at low frequencies, DBS caused the oscillation frequency to decrease and the oscillations to occur in bursts. This may also be consistent with findings that low-frequency DBS intensifies pathological activity, particularly as pathological neural activity in PD has been shown to contain more bursting mode for example in the STN (Bergman et al., 1994; Hassani et al., 1996; Kreiss et al., 1997). There is also evidence for both increases and decreases in burst activity in the GPi and GPe with DBS, as shown by Hahn et al., 2008 *via* STN DBS in MPTP monkeys. Furthermore, Hashimoto et al. (2003) showed that both low (2 Hz) and high-frequency stimulation lead to a bimodal firing rate distribution with high-frequency activity (ISI of 4 ms or 8 ms, i.e., 125 Hz or 250 Hz). Once again thalamic DBS and GPi DBS showed a similar pattern, but DBS to the GPe did not. Recent work has shown that GPi DBS has a similar effect on beta oscillations as STN DBS (Malekmohammadi et al., 2018). There are no accounts of the effect of human GPe DBS on beta-band activity in the brain, and while the presence of such oscillations can also be an aspect of healthy physiological brain activity, this demonstrates the ability of DBS *via* STN, GPi or thalamus to suppress beta frequency oscillations, and hypothesizes that GPe DBS may act *via* a different mechanism as previously suggested (Vitek et al., 2012). Hence, our predictions about the beta band activity and DBS would require testing *via* simultaneous stimulation and recording.

In conclusion, we have found that a single model of the basal ganglia, the thalamocortical network is able to support multiple types of oscillatory activity, which have been hypothesized to represent both healthy brain states and pathological neural activity. Our model, which abstracts out the details of a neuronal network and focusses solely on the dynamics of the connected populations, provides a basis for which to test the parameter space and therefore understand which connections play the main role in supporting and changing oscillatory activity. In particular, we show that the ascending connections are central for all types of oscillations, that the STN-GPe connection can switch the frequency band of oscillatory activity as could the balance of ascending and descending inputs to the thalamus. Finally, we show how high-frequency DBS changes the high amplitude oscillations in both tremor and beta band to a low-amplitude high-frequency activity, regardless of whether the thalamus or STN. Such work can help to understand the role of different brain regions and the mechanism by which DBS achieves a therapeutic effect. We propose that future work would involve combining such an approach with detailed single neuron models of the DBS target regions.

## Data Availability Statement

The code generated for this study is available from the ModelDB website (accession number 261882).

## Author Contributions

All authors contributed to the conception and design of the study. NY performed the simulations and analysis and wrote the first draft of the manuscript. All authors contributed to manuscript revision, read and approved the submitted version.

## Conflict of Interest

The authors declare that the research was conducted in the absence of any commercial or financial relationships that could be construed as a potential conflict of interest.
